# C3aR costimulation enhances the antitumor efficacy of CAR-T cell therapy through Th17 expansion and memory T cell induction

**DOI:** 10.1186/s13045-022-01288-2

**Published:** 2022-05-21

**Authors:** Peilong Lai, Xiaomei Chen, Yulian Wang, Jinghua Wang, Yuchen Zhang, Suxia Geng, Peng Li, Xin Du, Jianyu Weng, Duanqing Pei

**Affiliations:** 1 Guangzhou Institutes of Biomedicine and Health, Chinese Academy of Sciences, Guangzhou, 510530 China; 2 University of Chinese Academy of Sciences, Beijing, 100049 China; 3 Department of Hematology, Guangdong Provincial Hospital, Guangdong Academy of Medical Sciences, Guangzhou, 510080 China; 4 Laboratory of Cell Fate Control, School of Life Sciences, Westlake University, Hangzhou, 310024 China

**Keywords:** C3aR, Chimeric antigen receptor-modified T cell, Extramedullary leukemia, Leukemia, Multiple myeloma

## Abstract

Although chimeric antigen receptor (CAR)-modified adoptive T cell therapy is a promising immunotherapy for hematological malignancies, the efficacy improvement in relapsed/refractory acute lymphoblastic leukemia (ALL) with extramedullary infiltration and in multiple myeloma (MM) is still warranted. Since C3aR activation can promote the expansion of tumor-killing Th17 cells, we hypothesized that incorporating C3aR as a costimulatory domain would augment the antitumor activity of CAR-T. In this study, we introduced the C3aR domain into a CAR and generated BB-ζ-C3aR CAR-T targeting CD19 or BCMA. These new CAR-T exhibited a potent cytolytic ability to eradicate tumor cells expressing CD19 or BCMA in vitro. When administered intravenously to ALL or MM xenograft mouse models, BB-ζ-C3aR CAR-T reduced the tumor burden and improved the survival rate. Of note, these CAR-T could effectively eradicate subcutaneous CD19^+^ tumor cells, highlighting the therapeutic potential in extramedullary leukemia. Mechanistically, BB-ζ-C3aR CAR-T tended to exhibit a Th17 phenotype favoring tumor killing and suppressed Tregs. In addition, the induction of memory T cell in the BB-ζ-C3aR CAR-T cells indicated their long-term effects. Together, our findings suggest that the application of C3aR costimulation boosts the ability of CAR-T to eradicate aggressive tumor cells via Th17 expansion and memory T cell induction.

**Supplementary Information:**

The online version contains supplementary material available at 10.1186/s13045-022-01288-2.

**To the Editor:**

The genetic engineering of T cell to express chimeric antigen receptors (CAR) is recognized as a promising approach for hematological malignancies, but the effects of CAR-T cell therapy on relapsed/refractory acute lymphoblastic leukemia (ALL) with extramedullary infiltration and on multiple myeloma (MM) are limited and need to be improved [1–3]. The costimulatory molecule domains in CAR are required for the activation, expansion, and survival of CAR-T. Currently, the optimal costimulatory molecules are still under investigation [4, 5]. C3aR, the receptor that recognizes the complement fragment C3a, not only mediates innate immune responses but also participates in the induction of T cell responses [6–8]. Thus, we introduced the C3aR domain (Additional file 1: Fig. S1a) to the 3’ end of CD3ζ, which followed the 4-1BB domain, to generate a novel type of BB-ζ-C3aR CAR.

To evaluate the efficacy of T cell bearing this new CAR in ALL or MM, an anti-CD19 scFv or anti-BCMA scFv was included in the CAR (Additional file 1: Fig. S1b-e). Initially, we detected the activity of 19-BB-ζ-C3aR CAR-T in vitro. They showed stronger cytotoxicity to tumor cells than did 19-BB-ζ controls (Fig. 1a). In vivo, NOD-SCID-IL2rg^−/−^ (NCG) mice received an intravenous injection of NAML6-Luc cells, followed by treatment with CAR-T (2 × 10^6^ cells intravenously administered on D2 and D8). Then, the mice were examined by serial bioluminescence imaging (BLI) (Fig. 1c). As expected, the effect of 19-BB-ζ-C3aR CAR-T was more pronounced in tumor eradication, with a better survival rate achieved than in those mice treated with the 19-BB-ζ control (Fig. 1d–f). Furthermore, lowest expression of PD-1 and fewest CD19^+^ blasts were found in the 19-BB-ζ-C3aR CAR-T cell group, whereas no differences were observed in the numbers of GFP^+^ CAR-T cell, CD4^+^ and CD8^+^ T cell among three groups (Additional file 2: Fig. S2; Fig. 1g–j).

**Fig. 1:**
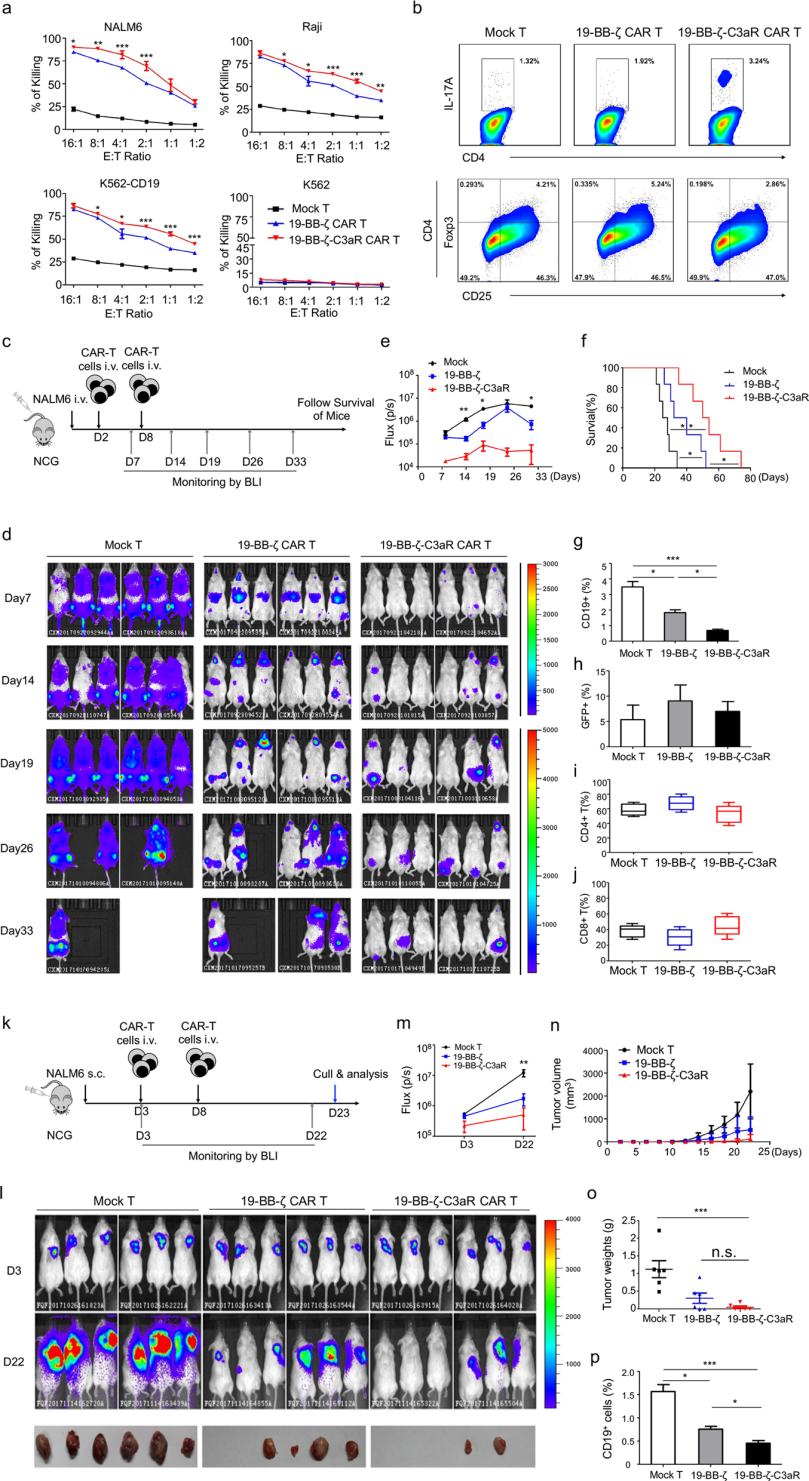
19-BB-ζ-C3aR CAR-T cells displayed potent anti-leukemia activity in vitro and in vivo, particularly in the xenografts extramedullary leukemia model. **a** The 19-BB-ζ-C3aR CAR-T cells showed significantly increased ability to lyse CD19-expressing tumor cells compared to 19-BB-ζ CAR-T cells. The cytotoxicity assay was performed at least three independent experiments. **b** Flow-cytometry results revealed enhanced expansion of Th17 cells and reduced Tregs in the 19-BB-ζ-C3aR CAR-T cells compared to 19-BB-ζ and mock-transduced T cells. **c** To establish the ALL model, 5 × 10^5^ NAML6-Luc cells were administered intravenously into NCG mice, which were randomized to the treatment with 2 × 10^6^ indicated T cell on Day 2 and Day 8. NAML6 tumor growth was then monitored by Xenogen imaging. **d** Bioluminescence images of NCG mice at Days 7, 14, 19, 26, and 33 are depicted for each group. **e** The curve of flux on indicated time points. **f** Kaplan–Meier survival analysis for ALL mice. Log-rank tests were used to perform statistical analyses of survival between groups. **g** The 19-BB-ζ-C3aR CAR-T group showed significantly fewer blast counts than Mock and 19-BB-ζ CAR-T groups. **h** The detectable GFP-positive T cells were similar in three groups. **i**, **j** There were no differences in CD4^+^ and CD8^+^ T cells between the indicated T cell populations. **k** Xenograft extramedullary leukemic model was established by subcutaneous injection of 5 × 10^5^ NALM-6 cells. The indicated CAR-T with 2 × 10^6^ dose were intravenously injected on Day 3 and Day 8, respectively. **l** NAML6 subcutaneous tumor growth was monitored by Xenogen imaging. **m** The curve of flux on indicated time points. **n**, **o** The tumor mass and weight were measured and recorded. **p** The 19-BB-ζ-C3aR CAR-T group showed the lowest CD19^+^ ALL blast counts on Day 22. ***p ≤ 0.001; **p ≤ 0.01; *p ≤ 0.05, n.s. no significant

Notably, to validate the potential of 19-BB-ζ-C3aR CAR-T in eradicating extramedullary leukemic cells, a subcutaneous leukemia mouse model was established by subcutaneously injecting 5 × 10^5^ NALM-6 cells (Fig. 1k). Strikingly, 19-BB-ζ-C3aR CAR-T significantly suppressed the subcutaneous tumor growth (Fig. 1i–m). Although no significant differences were found in tumor volume or weight between both CAR-T cell groups (Fig. 1m, o), more mice treated with 19-BB-ζ-C3aR CAR-T cells achieved complete tumor regression (Fig. 1l) and had fewer CD19-expressing tumor cells (Fig. 1p), highlighting their potent efficacy in extramedullary leukemia.

Similarly, BB-ζ-C3aR CAR-T targeting the BCMA antigen displayed better activity and efficacy than BCMA-BB-ζ CAR-T in vitro (Additional file 3: Fig. S3a). In vivo, MM-bearing mice receiving BCMA-BB-ζ-C3aR CAR-T showed the lowest tumor burden (Fig. 2a–c) and the longest survival time (Fig. 2d) with the fewest BCMA-expressing tumor cells (Fig. 2e). Thus, BB-ζ-C3aR CAR-T cells targeting BCMA possessed potent antitumor activity against MM.

**Fig. 2:**
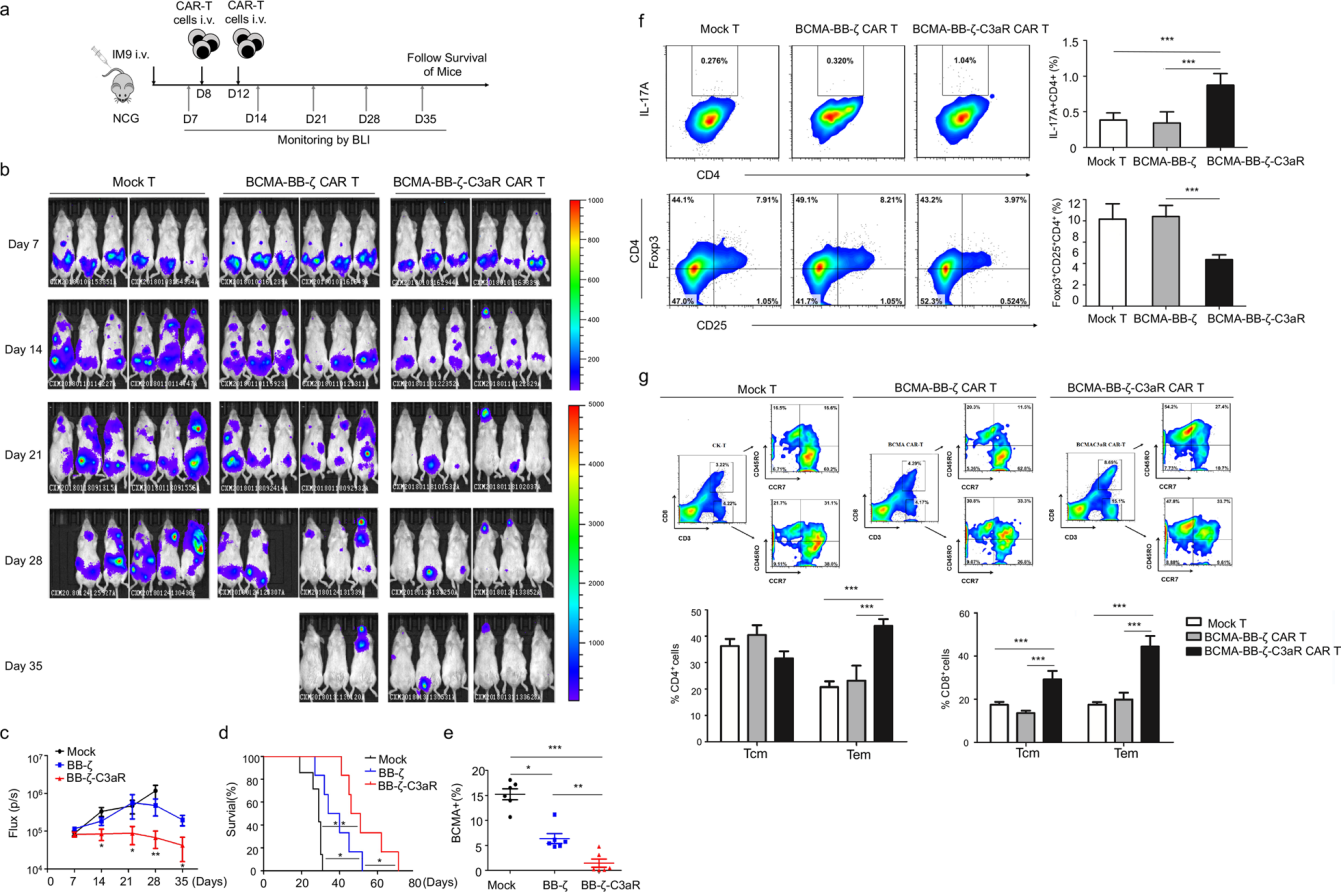
The BB-ζ-C3aR CAR-T cells significantly eradicated BCMA-expressing tumor cells through favoring Th17 cells expansion and memory T cells induction. **a** IM9-Luc cells with 5 × 10^5^ dose were administered intravenously into NCG mice to establish the MM model. These mice were randomized to the treatment of 2 × 10^6^ indicated T cell on Day 8 and Day 12. IM9 tumor growth was then monitored by Xenogen imaging. **b** Bioluminescence images of MM mice on Days 7, 14, 21, 28, and 35 are depicted for each group. **c** The curve of flux on indicated time points. **d** Kaplan–Meier survival analysis showed the longest time of survival in BCMA-BB-ζ-C3aR CAR-T cells group. **e** Hardly any BCMA^+^ tumor cells were detected in peripheral blood from mice treated with BCMA-BB-ζ-C3aR CAR-T cells. **f** In the xenograft MM mice, the BCMA-BB-ζ-C3aR CAR-T cells promoted the generation of IL-17-expressing Th17 cells and reduced the Tregs compared to the BCMA-BB-ζ CAR-T cells group. **g** A fraction of CD4^+^ or CD8^+^ cells exhibited the features of central memory cells (Tcm) with notably high expression of CCR7 and CD45RO. Tcm cells in CD8^+^ compartment were increased in the BCMA-BB-ζ-C3aR CAR-T-treated mice compared to BCMA-BB-ζ controls. In addition, the percentage of CD45RO^+^CCR7^−^ effector memory cells (Tem) was significantly increased in both CD4^+^ and CD8^+^ compartments in the BCMA-BB-ζ-C3aR CAR-T cells. ***p ≤ 0.001; **p ≤ 0.01; *p ≤ 0.05, n.s. no significant

Mechanistically, we found that C3aR incorporation improved the generation of Th17 cells while suppressing the differentiation of Tregs (Fig. 1b, Additional file 4: Fig. S4a, b). Consistently, the BB-ζ-C3aR CAR-T produced high level of IL-17, IL-22, GM-CSF, and IP-10 (Additional file 5: Fig. S5). Importantly, IL-17A blockade by secukinumab could abolish the cytotoxicity of 19-BB-ζ-C3aR CAR-T cells, indicating that IL-17A/Th17 was required for the tumor eradication process (Additional file 6: Fig. S6). In vivo, both the ALL and MM models exhibited an increase in Th17 cell and a decrease in Tregs after BB-ζ-C3aR CAR-T cell administration (Additional file 4: Fig. S4c, Fig. 2f), indicating that C3aR incorporation induced CAR-T to adopt the Th17 phenotype instead of differentiating into Tregs. In addition, we assessed memory T cell subsets to evaluate the persistence of CAR-T. In both the CD4^+^ and CD8^+^ compartments, enrichment of T central memory cells (Tcm) was observed in the 19-BB-ζ-C3aR group (Additional file 4: Fig. S4d). BCMA-BB-ζ-C3aR CAR-T cell treatment also presented increases in T central memory cells (Tem) and Tcm cells (Fig. 2g), suggesting that C3aR incorporation promoted the memory function of CAR-T.

In summary, we reported that C3aR, a novel costimulatory domain, significantly enhanced the antitumor ability of CAR-T and specifically improved therapeutic efficacy in extramedullary leukemia. BB-ζ-C3aR CAR-T promoted tumor eradication with long-term effects through Th17 expansion and memory T cell induction. These results not only highlight the importance of optimizing CAR engineering but also provide evidence that BB-ζ-C3aR CAR-T cells may be effective in treating refractory tumors, such as extramedullary leukemia and solid tumors.

## Supplementary Information

**Additional file 1: Fig. S1.** Generation of the BB-ζ-C3aR CAR-T cells targeting CD19 or BCMA. **a** The amino acid sequence of incorporated C3aR domain. **b** Schematic representation of chimeric antigen receptors that contain C3aR as a costimulation signal associated with 4-1BB in the intracellular domains, CD19-scFv, and the CD3ζ signal transduction domain. **c** Transduction efficiency of T cell was determined by flow cytometry. Representative results are from at least three independent experiments. **d** Schematic representation of chimeric antigen receptors that contain the C3aR as a costimulation signal associated with 4-1BB in the intracellular domain, BCMA-scFv, and the CD3ζ signal transduction domain. **e** Transduction efficiency of T cell was determined by flow cytometry.

**Additional file 2: Fig. S2.** The expression of PD-1 was reduced in the 19-BB-ζ-C3aR CAR-T cells. The expression of PD-1 on T cell was determined by flow cytometry. The results showed that BB-ζ-C3aR CAR-T cells presented lower expression of PD-1 compared with mock T or BB-ζ CAR-T cells. *p ≤ 0.05.

**Additional file 3: Fig. S3.** BCMA-BB-ζ-C3aR CAR-T cells exhibited potent anti-tumor activity in vitro. **a** The results of cytotoxicity assay showed that BCMA-BB-ζ-C3aR CAR-T cells have improved the ability to lyse BCMA^+^ MM cells compared to BCMA-BB-ζ CAR-T cells. **b** Flow cytometry and the statistics revealed that BCMA-BB-ζ-C3aR CAR-T cells treatment group showed more IL-17-expressing Th17 cells and less CD4^+^CD25^+^FoxP3^+^ Tregs compared to BCMA-BB-ζ and mock-transduced T cells group. ***p ≤ 0.001, **p ≤ 0.01, *p ≤ 0.05.

**Additional file 4: Fig. S4.** C3aR incorporation induced CAR-T cells to display phenotypes of Th17 and memory T cells in vivo. The statistics of IL-17-expressing Th17 cells (**a)** and CD4^+^CD25^+^FoxP3^+^ Tregs **(b)** in the co-culture system of CAR-T and CD19 expressing tumors. **c** In the xenograft leukemic mice, the 19-BB-ζ-C3aR CAR-T cells exhibited elevated expansion of Th17 cell phenotype. **d** In the 19-BB-ζ-C3aR CAR-T group, Tcm cells were highly induced in CD4^+^ and CD8^+^ T cells compared to those from 19-BB-ζ CAR-T group. No difference was observed in the percentage of Tem cells between both groups. ***p ≤ 0.001, **p ≤ 0.01, *p ≤ 0.05.

**Additional file 5: Fig. S5.** 19-BB-ζ-C3aR CAR-T presented with an expression elevation of some cytokines. In vitro, increased expressions of IL-17, IL-22, GS-CSF, and IP-10 were observed in the 19-BB-ζ-C3aR CAR-T, whereas no differences in TNF-a and IFN-r were found between 19-BB-ζ-C3aR CAR-T and 19-BB-ζ CAR-T. **p ≤ 0.01, *p ≤ 0.05, n.s. no significant.

**Additional file 6: Fig. S6.** IL-17A blockade by secukinumab impaired the tumor eradication effect of 19-BB-ζ-C3aR CAR-T. In vitro, secukinumab, a human IgG1κ monoclonal antibody that binds to the IL-17A, suppressed the cytotoxicity of 19-BB-ζ-C3aR CAR-T on CD19-expressing NALM6 cells. ***p ≤ 0.001, **p ≤ 0.01.

**Additional file 7.** Materials and Methods.

## Data Availability

All data generated or analyzed during this study are included in this published article or its supplementary information files. The raw datasets are available from the corresponding authors on reasonable request.

## Abbreviations

ALL: Acute lymphoblastic leukemia
BLI: Bioluminescence imaging
CAR: Chimeric antigen receptor
CAR-T: Chimeric antigen receptor T cell
C3aR: Complement C3aR
i.v.: Intravenous
MM: Multiple myeloma
NCG: NOD-SCID-IL2rg^−^^/^^−^
Tcm: T central memory cells
Tem: T effector memory cells
Tregs: Regulatory T cells
