# Supplementary figures and images for: C3aR costimulation enhances the antitumor efficacy of CAR-T cell therapy through Th17 expansion and memory T cell induction
Source: J Hematol Oncol. 2022 May 21;15:68. doi: 10.1186/s13045-022-01288-2 (PMC9124432; doi:10.1186/s13045-022-01288-2)

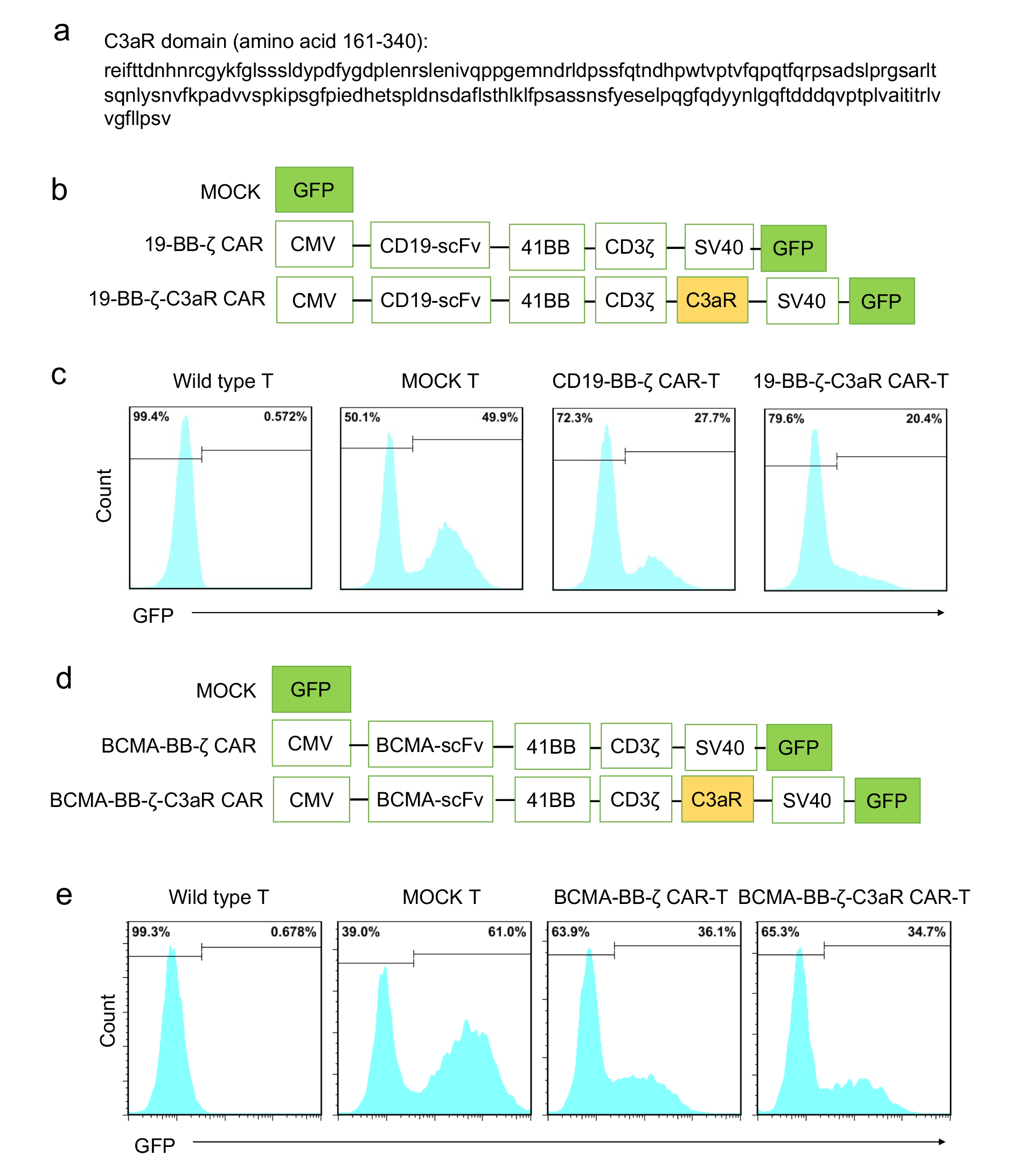

Supplement: Supplementary file 1 — Additional file 1: Fig. S1. Generation of the BB-ζ-C3aR CAR-T cells targeting CD19 or BCMA. a The amino acid sequence of incorporated C3aR domain. b Schematic representation of chimeric antigen receptors that contain C3aR as a costimulation signal associated with 4-1BB in the intracellular domains, CD19-scFv, and the CD3ζ signal transduction domain. c Transduction efficiency of T cell was determined by flow cytometry. Representative results are from at least three independent experiments. d Schematic representation of chimeric antigen receptors that contain the C3aR as a costimulation signal associated with 4-1BB in the intracellular domain, BCMA-scFv, and the CD3ζ signal transduction domain. e Transduction efficiency of T cell was determined by flow cytometry. [file 13045_2022_1288_MOESM1_ESM.tif]

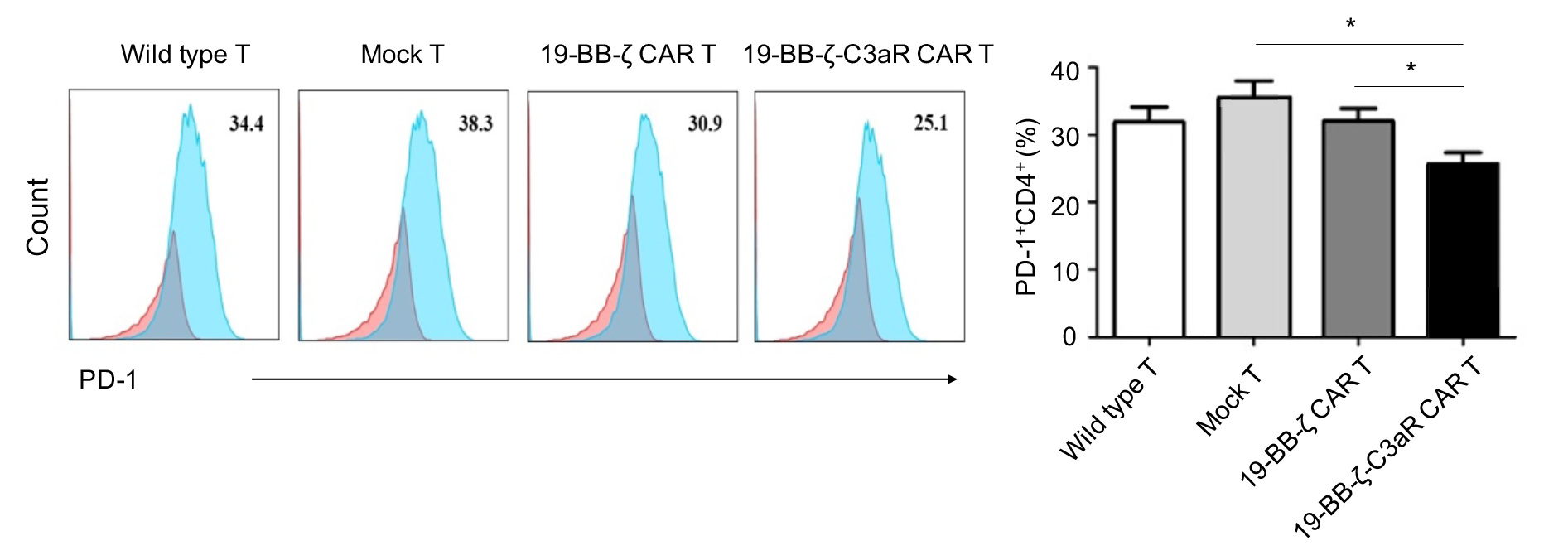

Supplement: Supplementary file 2 — Additional file 2: Fig. S2. The expression of PD-1 was reduced in the 19-BB-ζ-C3aR CAR-T cells. The expression of PD-1 on T cell was determined by flow cytometry. The results showed that BB-ζ-C3aR CAR-T cells presented lower expression of PD-1 compared with mock T or BB-ζ CAR-T cells. *p ≤ 0.05. [file 13045_2022_1288_MOESM2_ESM.tif]

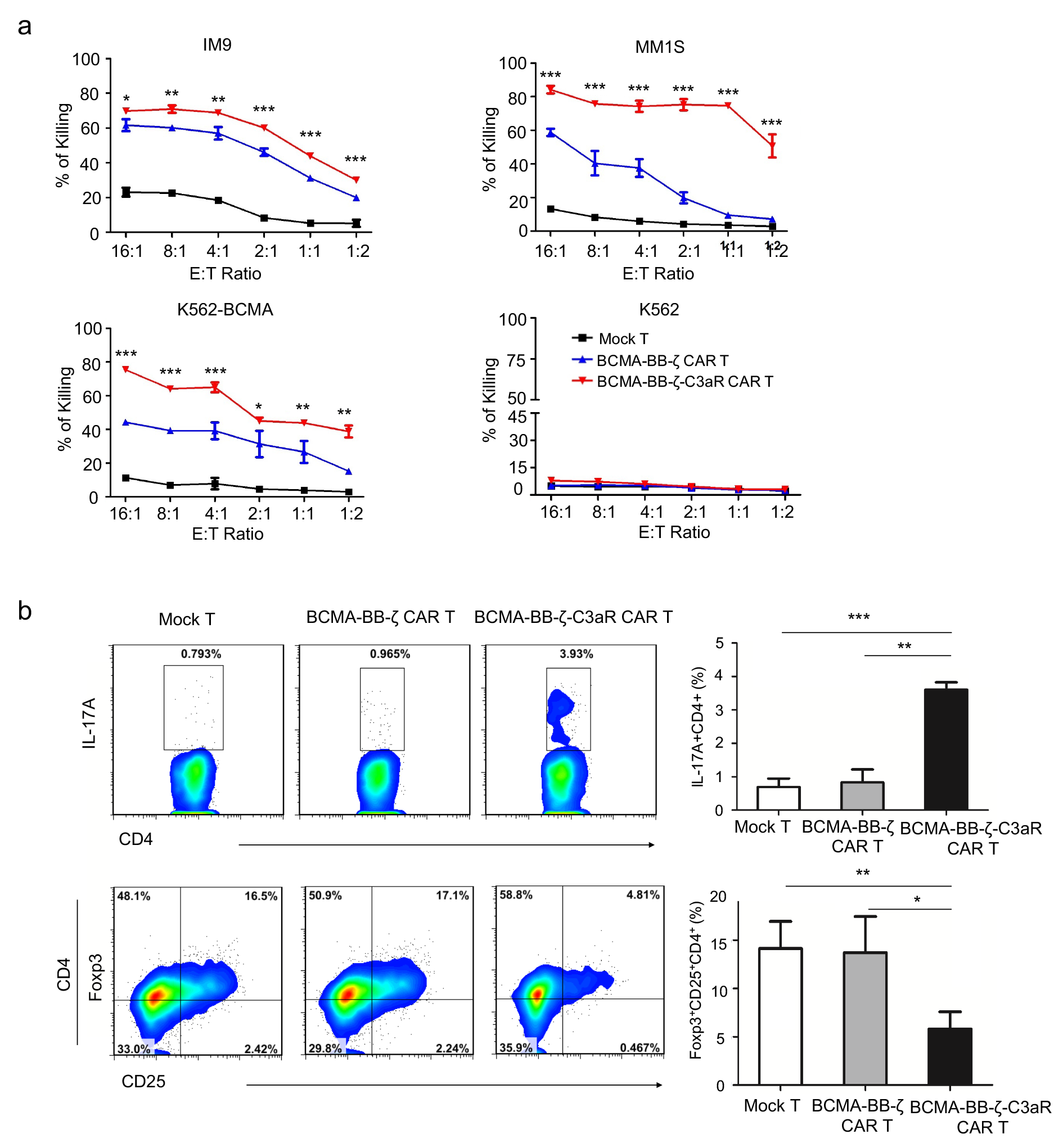

Supplement: Supplementary file 3 — Additional file 3: Fig. S3. BCMA-BB-ζ-C3aR CAR-T cells exhibited potent anti-tumor activity in vitro. a The results of cytotoxicity assay showed that BCMA-BB-ζ-C3aR CAR-T cells have improved the ability to lyse BCMA+ MM cells compared to BCMA-BB-ζ CAR-T cells. b Flow cytometry and the statistics revealed that BCMA-BB-ζ-C3aR CAR-T cells treatment group showed more IL-17-expressing Th17 cells and less CD4+CD25+FoxP3+ Tregs compared to BCMA-BB-ζ and mock-transduced T cells group. ***p ≤ 0.001, **p ≤ 0.01, *p ≤ 0.05. [file 13045_2022_1288_MOESM3_ESM.tif]

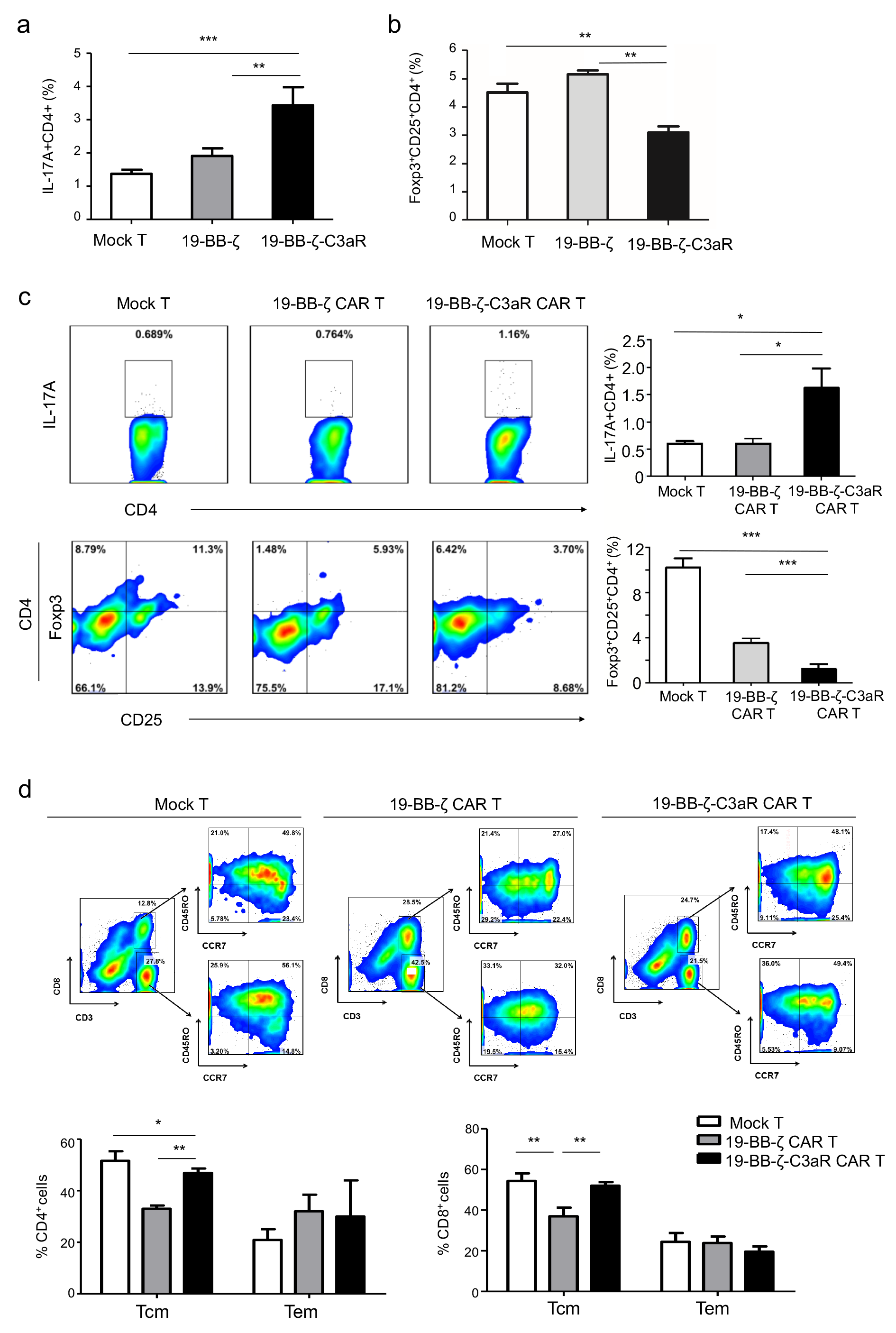

Supplement: Supplementary file 4 — Additional file 4: Fig. S4. C3aR incorporation induced CAR-T cells to display phenotypes of Th17 and memory T cells in vivo. The statistics of IL-17-expressing Th17 cells (a) and CD4+CD25+FoxP3+ Tregs (b) in the co-culture system of CAR-T and CD19 expressing tumors. c In the xenograft leukemic mice, the 19-BB-ζ-C3aR CAR-T cells exhibited elevated expansion of Th17 cell phenotype. d In the 19-BB-ζ-C3aR CAR-T group, Tcm cells were highly induced in CD4+ and CD8+ T cells compared to those from 19-BB-ζ CAR-T group. No difference was observed in the percentage of Tem cells between both groups. ***p ≤ 0.001, **p ≤ 0.01, *p ≤ 0.05. [file 13045_2022_1288_MOESM4_ESM.tif]

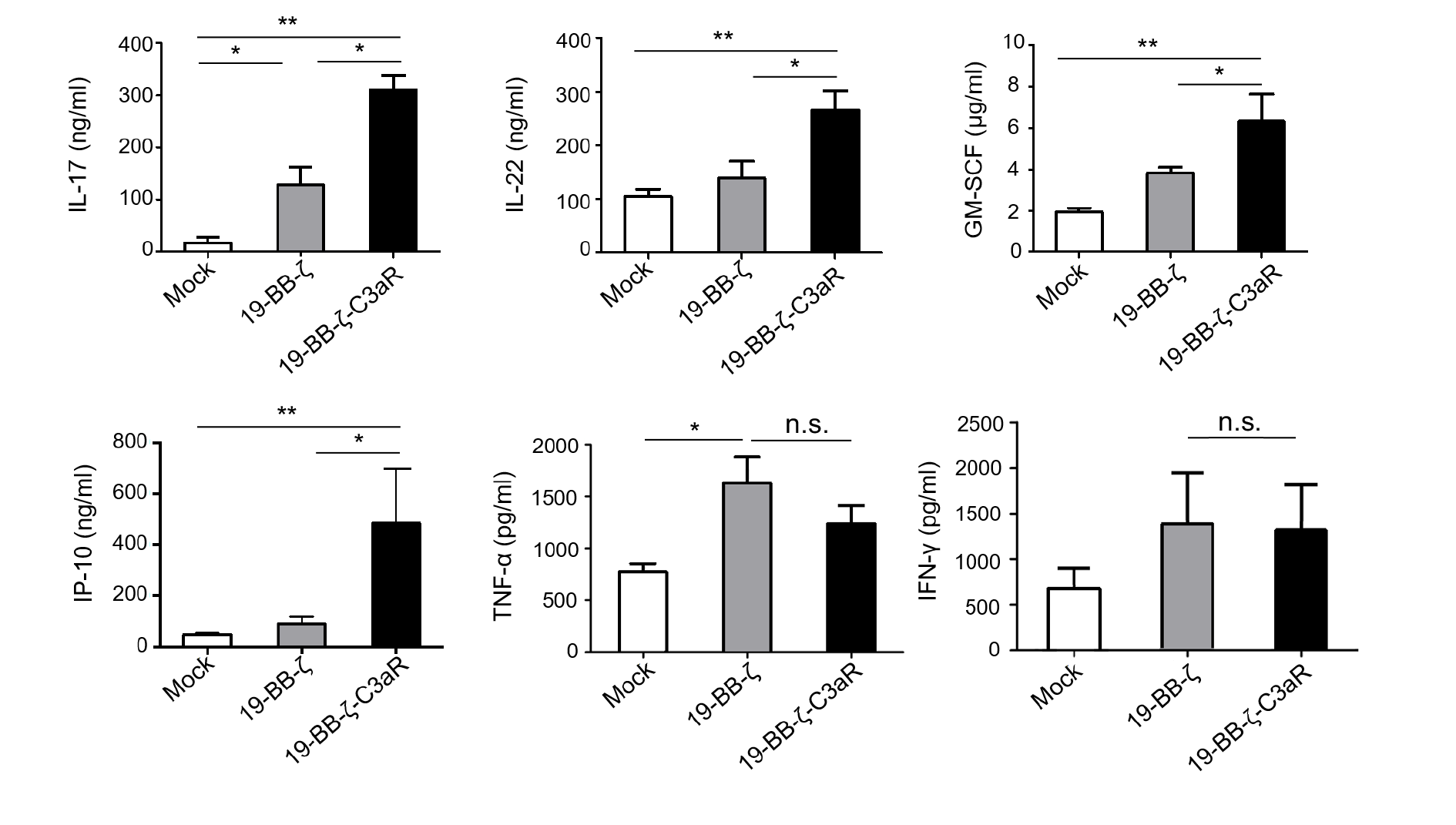

Supplement: Supplementary file 5 — Additional file 5: Fig. S5. 19-BB-ζ-C3aR CAR-T presented with an expression elevation of some cytokines. In vitro, increased expressions of IL-17, IL-22, GS-CSF, and IP-10 were observed in the 19-BB-ζ-C3aR CAR-T, whereas no differences in TNF-a and IFN-r were found between 19-BB-ζ-C3aR CAR-T and 19-BB-ζ CAR-T. **p ≤ 0.01, *p ≤ 0.05, n.s. no significant. [file 13045_2022_1288_MOESM5_ESM.tif]

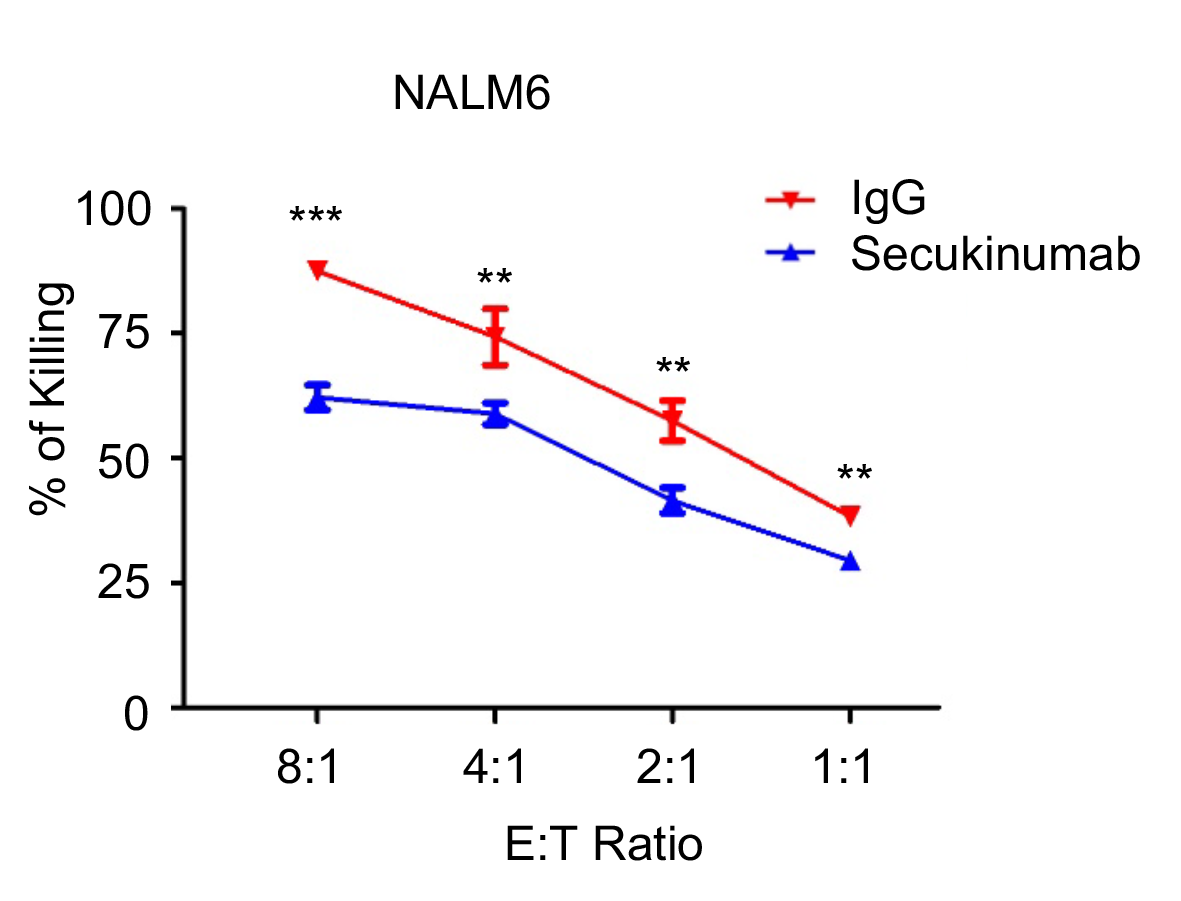

Supplement: Supplementary file 6 — Additional file 6: Fig. S6. IL-17A blockade by secukinumab impaired the tumor eradication effect of 19-BB-ζ-C3aR CAR-T. In vitro, secukinumab, a human IgG1κ monoclonal antibody that binds to the IL-17A, suppressed the cytotoxicity of 19-BB-ζ-C3aR CAR-T on CD19-expressing NALM6 cells. ***p ≤ 0.001, **p ≤ 0.01. [file 13045_2022_1288_MOESM6_ESM.tif]
