# Supplementary material for: C3aR costimulation enhances the antitumor efficacy of CAR-T cell therapy through Th17 expansion and memory T cell induction
Source: J Hematol Oncol. 2022 May 21;15:68. doi: 10.1186/s13045-022-01288-2 (PMC9124432; doi:10.1186/s13045-022-01288-2)
Supplement: Supplementary file 7 — Additional file 7. Materials and Methods. [file 13045_2022_1288_MOESM7_ESM.doc]

**Materials and Methods**

**Lentiviral vectors construction**

The GFP, CD19-BB-ζ, BCMA-BB-ζ, CD19-BB-ζ-C3aR, BCMA-BB-ζ-C3aR expressing lentiviral vectors were prepared as described^4^. The anti-CD19 or anti-BCMA CAR lentiviruses were constructed with 4-1BB, CD3ζ and C3aR domain (amino acid 161-340) in order. Firstly, the full length of the chimeric gene for CD19-BB-ζ or BCMA-BB-ζ was synthesized. Then overlapping PCR was done to attach the sequence of C3aR domain to the 3’ end of CD19-BB-ζ CAR or BCMA-BB-ζ CAR. The individual CAR was cloned into pLent-EF1a under the control of CMV promoter.

**Lentivirus manufacture**

The lentivirus was produced in HEK-293T cells via lipofectamine 3000 (Invitrogen, USA) transfection. The HEK-293 T cells were co-transfected with the pLent-EF1a-based lentiviral plasmid and two packaging plasmids of psPAX2 and pMD2.G. Lentivirus-containing supernatants were harvested 48h and 72 h after transduction and were filtered through a 0.45-μm filter.

**Generation and expansion of CAR-T**

The CAR-T cells were generated as previously described^2^. Briefly, mononuclear cells were separated from peripheral blood (PB) using Ficoll. CD3^+^ T-cell were isolated using human CD3 microbeads (Milteyni, USA) and then were cultured in RPMI 1640 medium supplemented with 10% FBS (Gibco, USA), 100-IU/mL penicilin and 100 IU/mL streptomycin sulfate. The T-cell were activated using human T-activator CD3/CD28 Dynabeads (Gibco, USA) at a cell-to-bead ratio of 1:1 for 48h. Then they were transfected with supernatant containing lentiviral vectors expressing GFP or different CARs. Twelve hours later, the medium was replaced with R10 supplemented with 300IU/mL IL-2 (PeproTech, USA). These T-cell were fed every two days with fresh media for expansion.

**Cells**

The cell lines of NALM6, RAJI, IM9, MM1S, K562 were obtained from the ATCC and maintained in R10 medium. Primary human ALL or MM cells were isolated from PB using Ficoll. HEK-293 T cells were maintained in Dulbecco’s modified Eagle’s medium (Gibco, USA). NALM6 and IM9 cells were transduced with GFP and luciferase for in vivo experiments. The K562-CD19 and K562-BCMA cell lines were generated through the transduction of K562 cells with a CD19-expressing lentiviral vector or BCMA-expressing lentiviral vector. CD19^+^ cells and BCMA^+^ cells were isolated using human CD19 microbeads or human BCMA microbeads (Milteyni, USA). All samples were obtained after informed, written consent from patients.

**Killing assays**

For the in vitro functional studies, the cell lines (NALM6, RAJI, IM9, MM1S, K562, K562-CD19, K562-BCMA cells) or primary blasts (CD19^+^ and BCMA^+^) were washed and re-suspended at 5×10^5^/mL. These cells were labeled with 5uL of carboxyfluorescein succinimidyl ester (CFSE) in 5uM (Gibco, USA) for 15 min at 37 °C. Secukinumab (10ug/mL), a human IgG1κ monoclonal antibody that binds to the IL-17A, was added in the in vitro co-culture system of NALM6 cells and 19-BB-ζ-C3aR CAR-T cells. The reaction was quenched with R10 media, and the cells were washed three times. These CFSE-labelled cells as target cells were incubated with effector T cells at the indicated ratios in triplicate wells in U-bottomed, 96-well plates. Twenty-four hours later, PI (BD) were added in cell suspensions and the residual live target cells were identified as CFSE^+^ PI^–^. Background luminescence was negligible (< 1% than the signal from the wells with only target cells). The viability percentage was calculated as experimental signal/maximal signal × 100%, and killing percentage was equal to 100% minus viability percentage.

**Xenograft models and in vivo assessment**

NCG (NOD-SCID-IL2rg^–/–^, Model Animal Research Center of Nanjing University, China) mice were used to establish the xenograft models. All mice were maintained in specific pathogen-free-grade cages and were provided with autoclaved food and water. The NCG mice received intravenous injection of 5×10^5^ NALM6-luc or IM9-luc cells in 300μL PBS to develop ALL or MM models. For subcutaneous tumor xenograft mouse model, 5×10^5^ NALM6-luc cells in 300μL of PBS were injected subcutaneously into the right flanks of NCG mice. At the indicated time, 2×10^6^ indicated T-cell in 300μL of PBS were adoptively transferred to tumor-bearing mice by tail vein injection.

**In vivo imaging**

In vivo whole-body imaging of luciferase-labeled cells was performed using a cooled CCD camera system (IVIS 100 Series Imaging System, Xenogen, Alameda, CA, USA). Mice were injected with D-luciferin firefly potassium salt at 75 mg/kg and imaged 5 minutes after the injection. Living Image software (Xenogen) was used to quantify total and average emissions.

**Flow cytometry**

The flow cytometry was performed to detect the GFP% of transduced T-cell, CD19^+^ and BCMA^+^ expression on leukemia cells, and T cell phenotypes of the CAR-T-cell. The PBMC, splenocytes and BM cells were isolated with a red blood cell lysis buffer (Biolegend), and were blocked with Fc Block (BD Pharmingen, San Diego, CA) for 15 minutes. The first antibodies were incubated for 20 minutes at room temperature. The antibodies included anti-CD3 (APC), anti-CD4 (Percp-cy5.5), anti-CD8 (PE), anti-CD19 (APC), anti-BCMA (APC), anti-CD25 (PE), anti-FoxP3 (APC), anti-IL-17A (PE), anti-PD-1 (PE), anti-CD45RO (APC) and anti-CCR7 (APC-CY7) (eBioscience, USA). FoxP3 and IL-17A intracellular staining were performed using an eBioscience kit according to the manufacturer’s protocol. All the cells were detected on a FACSCanto II flow cytometer (Becton Dickinson, USA) and the data were analyzed in FlowJo software (TreeStar).

**Statistics**

The statistical analysis was performed with SPSS software version 19.0 (Inc., Chicago, IL, USA). Student’s t test (two-tailed) for two groups or one-way analysis of variance (ANOVA) for three or more groups were used accordingly. The data were presented as means ± SEM. Survival curves were plotted as Kaplan-Meier curves and analyzed with log-rank tests. A *P* values < 0.05 were considered statistically significant (^***^p ≤ 0.001, ^**^p ≤ 0.01, ^*^p ≤ 0.05).
